# Computational Study of Potential Galectin-3 Inhibitors in the Treatment of COVID-19

**DOI:** 10.3390/biomedicines9091208

**Published:** 2021-09-13

**Authors:** Maral Aminpour, Marco Cannariato, Angelica Zucco, Elisabetta Di Gregorio, Simone Israel, Annalisa Perioli, Davide Tucci, Francesca Rossi, Sara Pionato, Silvia Marino, Marco A. Deriu, Kiran K. Velpula, Jack A. Tuszynski

**Affiliations:** 1 Department of Biomedical Engineering, University of Alberta, Edmonton, AB T6G 1Z2, Canada; aminpour@ualberta.ca; 2 DIMEAS, Politecnico di Torino, Corso Duca degli Abruzzi 24, 10129 Torino, Italy; marco.cannariato@studenti.polito.it (M.C.); s289475@studenti.polito.it (A.Z.); s277539@studenti.polito.it (E.D.G.); s275375@studenti.polito.it (S.I.); s285863@studenti.polito.it (A.P.); s269635@studenti.polito.it (D.T.); s285853@studenti.polito.it (F.R.); s289369@studenti.polito.it (S.P.); s279885@studenti.polito.it (S.M.); marco.deriu@polito.it (M.A.D.); 3 Department of Cancer Biology and Pharmacology, Pediatrics and Neurosurgery, University of Illinois College of Medicine at Peoria, Peoria, IL 61605, USA; 4 Department of Physics, University of Alberta, Edmonton, AB T6G 2E1, Canada

**Keywords:** Galectin-3, spike, dual inhibitors, COVID-19, docking

## Abstract

Galectin-3 is a carbohydrate-binding protein and the most studied member of the galectin family. It regulates several functions throughout the body, among which are inflammation and post-injury remodelling. Recent studies have highlighted the similarity between Galectin-3′s carbohydrate recognition domain and the so-called “galectin fold” present on the N-terminal domain of the S1 sub-unit of the SARS-CoV-2 spike protein. Sialic acids binding to the N-terminal domain of the Spike protein are known to be crucial for viral entry into humans, and the role of Galectin-3 as a mediator of lung fibrosis has long been the object of study since its levels have been found to be abnormally high in alveolar macrophages following lung injury. In this context, the discovery of a double inhibitor may both prevent viral entry and reduce post-infection pulmonary fibrosis. In this study, we use a database of 56 compounds, among which 37 have known experimental affinity with Galectin-3. We carry out virtual screening of this database with respect to Galectin-3 and Spike protein. Several ligands are found to exhibit promising binding affinity and interaction with the Spike protein’s N-terminal domain as well as with Galectin-3. This finding strongly suggests that existing Galectin-3 inhibitors possess dual-binding capabilities to disrupt Spike–ACE2 interactions. Herein we identify the most promising inhibitors of Galectin-3 and Spike proteins, of which five emerge as potential dual effective inhibitors. Our preliminary results warrant further in vitro and in vivo testing of these putative inhibitors against SARS-CoV-2 with the hope of being able to halt the spread of the virus in the future.

## 1. Introduction

The COVID-19 pandemic is a worldwide emergency, as its rapid spread and high mortality rate have caused severe challenges. On 30 January 2020, the World Health Organization declared the SARS-CoV-2 epidemic a public health emergency of international concern [1]. The number of people infected with severe acute respiratory syndrome coronavirus 2 (SARS-CoV2), the causative agent of COVID-19, has been steadily increasing worldwide [2]. SARS-CoV-2 is an enveloped non-segmented positive-sense RNA β-coronavirus belonging to the same family as the severe acute respiratory syndrome (SARS) and Middle East Respiratory Syndrome (MERS) viruses [3]. Most patients with COVID-19 exhibit mild to moderate symptoms, but approximately 15% progress to severe pneumonia, and about 5% eventually develop acute respiratory distress syndrome (ARDS), septic shock, and/or multiple organ failure. The focus of clinical treatment is on symptom management and oxygen therapy, with mechanical ventilation for patients with respiratory failure. Although several antiviral drugs are being actively tested, none of them has yet been specifically approved for COVID-19.

In addition to vaccine development and approaches that directly target the virus or block viral entry, treatments that address the immunopathology of the infection have become a major focus [4]. It typically takes more than 10 years for a new therapeutic agent to complete the journey from R&D initial discovery to approval for use. As such, repurposing known drugs is a preferable option as a more expeditious and cost-effective approach for accelerating the development of new therapies for COVID-19. In the balance of this section of the paper, we first outline the principal features and functions of Spike and Galectin-3 (Gal-3) proteins. We then discuss how Gal-3 protein relates to COVID-19 pathogenesis and why Gal-3 may be a promising target in the treatment of COVID-19. Finally, we discuss the prospect of the inhibitors against human galectins binding to the S1 N-terminal domain (NTD) of β-coronaviridae, given the structural similarities between Gal-3 and the NTD domain of Spike protein (S1-NTD).

### 1.1. Spike: Main Features and Functions

**Figure 1:**
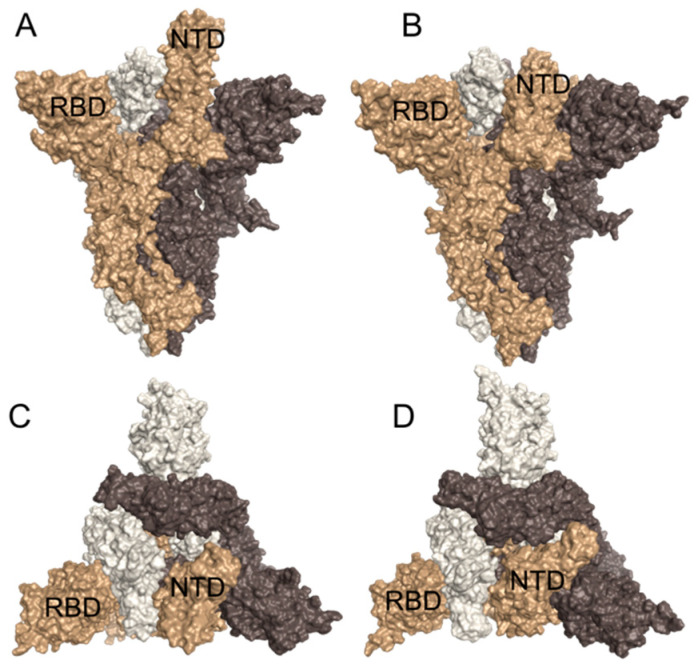
(**A**,**B**) Side view and (**C**,**D**) top view of Spike open (PDB: 6VSB) and Spike closed (PDB: 6VXX), respectively. Note that the NTD and RBD domains are labelled in all four panels.

The Spike protein (also called S-protein) component of SARS-CoV-2 is a central target in the fight against COVID-19 since it is the primary target of antibodies that provide immunity against the virus. The surfaces of coronaviruses are covered with these spikes, giving them their distinctive crown-like appearance in electron micrographs. The spikes initiate the process of infection, binding to receptors and then fusing with the cell membrane to release the viral genome inside. Many other enveloped viruses, including influenza hemagglutinin and the envelope glycoproteins of HIV-1 and Ebola, use similar spike-like proteins to infect cells. The spike protein is composed of three identical chains that together form a complex with a small domain inside the virus, a membrane-spanning segment, and a large ectodomain that extends outward from the virus. The S-protein exists in a metastable prefusion conformation that undergoes a substantial structural rearrangement to fuse the viral membrane with the host cell membrane. This process is triggered when the S_1_ sub-unit binds to a host cell receptor. Receptor binding destabilises the prefusion trimer, resulting in the shedding of the S_1_ sub-unit and transition of the S_2_ sub-unit to a stable post fusion conformation. To engage a host cell receptor, the receptor-binding domain (RBD) of S_1_ undergoes hinge-like conformational movements that transiently hide or expose the determinants of receptor binding. These two states are referred to as the “down” (or “closed”) conformation and the “up” (or “open”) conformation (Figure 1), where down corresponds to the receptor-inaccessible state and up corresponds to the receptor accessible state, which is presumed to be less stable [5].

**Figure 2:**
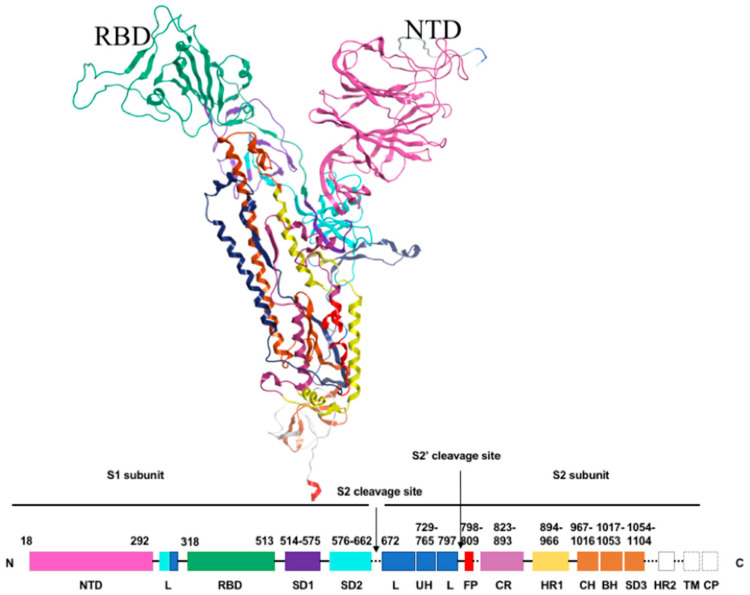
Schematic primary structure of the Spike Protein colored by domain. The NTD is represented by the residues 18-292.

The S_1_ sub-unit of the spike protein of SARS-CoV-2, critical for its entry into host cells, can be subdivided into an NTD and a C-terminal domain (CTD) (Figure 2). The role of the CTD in viral entry has been well characterised in the literature, as it binds angiotensin-converting-enzyme 2 (ACE2) receptors. 

ACE2 is an enzyme that activates angiotensin, a peptide hormone involved in the control of blood pressure. ACE2 is found on lung, heart, kidney, and intestinal cells, making these cells the primary targets for infection by the virus [5,6]. In addition, the spike is a glycoprotein: the ectodomain is covered with sugar chains that help to mask the virus from the immune system [6]. Watanabe et al. reported 22 N linked glycosylation sites on Spike protein. In their study, across the 22 N-linked glycosylation sites, 52% were found to be fucosylated, while 15% of the glycans contained at least one sialic acid residue. Sialic acids, it should be noted, have been described as “eccentric” in that they bind several different pathogens and toxins. Owing to their location and abundant distribution, sialic acids participate in a wide variety of physiological and pathological processes. Sialic acids are a common constituent of glycoproteins, glycolipids, and gangliosides. They decorate the terminal of sugar chains at the surface of cells or soluble proteins. Sialic acids linked to glycoproteins and gangliosides are recruited by a broad spectrum of viruses—including coronaviruses—as receptors and/or attachment factors for cell entry. In many viral infections (influenza, Ebola, SARSCoV, among others), glycan-mediated interactions are essential for the initial contact between the virus and the host [7,8,9]. The S_1_-NTD of SARS-CoV-2 has been shown to possess a sialic acid binding site highly similar to that observed in MERS-CoV [10,11]. In MERS-CoV, depletion of sialic acids inhibits cell entry by the virus. As such, the interaction between S_1_ -NTD and host sialic acids may be critical for SARS-CoV-2 cell entry as a means of stabilizing the interaction between the S_1_ -CTD and ACE2. 

### 1.2. Galectin-3: Main Features and Functions

Gal-3 is a carbohydrate-binding protein and the most studied member of the galectin family. In mammals, 15 galectins have been identified to date containing either one or two carbohydrate recognition domains (CRDs). Gal-3 is the sole member of the chimera-type family of galectins, with a single CRD and an intrinsically disordered sequence at the NTD that promotes oligomerization. Gal-3 (MW = 31 kDa) is found in solution as a monomer with two functional domains: an extra-long and flexible N-terminal domain, involving 100–150 amino acid residues, and a C-terminus, i.e., the CRD, consisting of about 135 amino acid residues. Gal-3 is predominantly located in the cytoplasm, where its synthesis occurs on free ribosomes, but it has also been detected in the nucleus, on the cell surface, and in the extracellular environment, suggesting a multifunctionality of this molecule [12]. It is most common in myeloid cells (macrophages, dendritic cells, neutrophils, and monocytes), epithelial cells, endothelial cells, and fibroblasts [13]. Moreover, Gal-3 regulates several functions throughout the body, including cell migration, adhesion, proliferation and differentiation, cell–cell and cell–matrix interactions, inflammation, modulation of apoptosis, angiogenesis, tumorigenesis, and post-injury remodelling [12,13]. Given this, it is not surprising that this protein is involved in the pathogenesis of many relevant human diseases, including cancer, fibrosis, chronic inflammation, and scarring affecting many different tissues [12]. The role of Gal-3 in physiological and pathological processes has prompted the development of several galectin inhibitors, some as novel therapeutics and others as experimental tools for basic science [14]. The Gal-3 inhibitors developed have ranged from the natural binding lactose motif to synthetic derivatives, such as monogalactosides and thiodigalactosides adorned with featuring non-carbohydrate structural elements such as aryl triazoles and aromatic amides [15]. The Galectin-3 protein is linked to COVID-19 pathogenesis in particular in terms of its role in Cytokine Storm Syndrome (CSS) and as a mediator of lung fibrosis [13].

#### 1.2.1. Cytokine Storm Syndrome

The major cause of death in patients infected with SARS-CoV2 is CSS. CSS develops due to hyper-activation of macrophages, monocytes, and dendritic cells, which are stimulated to release a variety of pro-inflammatory mediators including interleukin (IL)-1, IL-6, and tumour necrosis factor α (TNF-α). This in turn leads to systemic organ dysfunction that may result in death. Recent studies have shown that Gal-3 appears to be elevated in proliferative T cells in severe cases of COVID-19, and often the hyperinflammation phase involves the concomitant upregulation of Gal-3, TNF-α, and IL-6. Gal-3 can also act as a modulator of cytokine expression on the part of immune cells. Elevated levels of Gal-3 have been observed in the serum of patients suffering from severe COVID-19 as compared to those with mild disease. In particular, the highest levels of Gal-3 have been observed in infected macrophages, monocytes, and dendritic cells, the very cells responsible for initiating CSS [13]. Inhibition of Gal-3 significantly reduces the levels of these cytokines, and in this respect is promising as an agent for reducing the inflammatory response associated with COVID-19. 

#### 1.2.2. Gal-3 as a Mediator of Lung Fibrosis

Finally, it is well known that persistent viral infections are a risk factor for the subsequent development of pulmonary fibrosis. Gal-3 has already been tested as a therapeutic target in Idiopathic Pulmonary Fibrosis (IPF) with very promising results. Moreover, many studies have highlighted the importance of hypoxia-induced Gal-3 in PAH and lung function and, notably, cardiovascular disease, diabetes, and pneumonia are among the main risk factors for severe COVID-19 patients, all having in common elevated levels of Gal-3. The secretion of Gal-3 by macrophages upregulates transforming growth factor ß (TGF-ß) receptors on fibroblasts and myofibroblasts. This in turn activates these cells, initiating the formation of granulation tissue (via collagen deposition) that is eventually remodelled to a fibrous scar. In this regard, Gal-3 inhibitors show promise in limiting fibrotic change following lung injury. Moreover, influenza A virus and Streptococcus pneumonia also bind to Gal-3, increasing their airway epithelial adhesion, and thereby supporting the theory that Gal-3 plays an important role in primary and secondary airway infections among COVID-19 patients [16].

Other receptors, such as CD26 and CD147, have been proposed to interact with viral-spike protein, as they are known to bind to Gal-3. As such, Gal-3 inhibition is potentially life-saving for critically ill COVID-19 patients. In other words, Gal-3 could be a good prognostic marker for severe COVID-19, as elevated plasma levels of Gal-3 may be a trigger of the cytokine storm phenomenon observed in severe COVID-19 patients [16]. In this regard, Caniglia et al. proposed Gal-3 inhibitors as a potentially viable treatment to mitigate the entry of SARS-CoV2 and the inflammatory response associated with infection [13].

### 1.3. Sars-Cov2 Attachment: Implications of the Galectin-Like S1-NTD

Chiodo et al. [17] have suggested the existence of lectin-mediated molecular pathways that may play a role in viral infection and immune exacerbation, identifying some lectins that bind to the RBD. Most of the studies in this domain have focused on the RBD fragment of Spike protein. In a recent study, Lenza et al. [18] applied NMR experiments to study glycan-mediated interactions of the RBD fragment of Spike protein with a variety of human lectins using C-labelled glycans at N331 and N343 in the RBD domain of the Spike protein. In another study, Cai et al. [19] showed that GRFT, a lectin isolated from the red alga *Griffithsia sp*, can inhibit SARS-CoV-2 infection by binding to the glycosylation sites in the S1 sub-unit—possibly the RBD—of Spike protein. 

Besides RBD, there is an emergent and developing interest in NTD—the other component of the extracellular portion of the Spike protein—as another promising target for drug repurposing and vaccine and novel drug development [20,21,22,23]. One of the advantages of the NTD domain as a therapeutic target, it should be noted, is that the mutations identified in most of the variants to date have been located in the RBD domain. As such, i.e., in light of the composition of the variants identified, NTD appears to be a more stable target than RBD for drug development.

**Figure 3:**
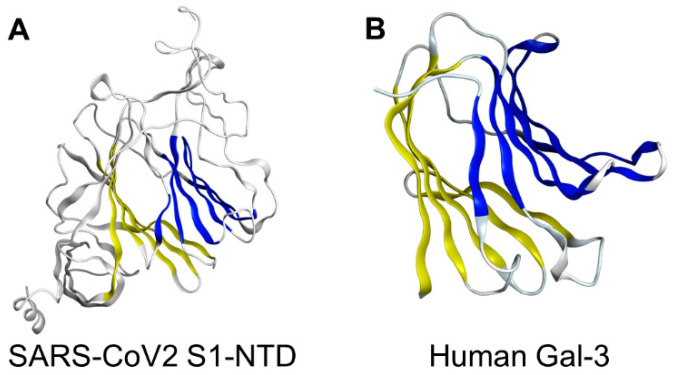
Structural similarities of SARS-CoV2 S1-NTD and human Gal-3. The structural topologies of the (**A**) SARS-CoV2 S1-NTD (PDB ID: 6VXX) and (**B**) human Gal-3 (PDB ID: 1KJR) are shown as schematic illustrations, where corresponding structures are depicted with the same colour and sialic acid binding site in SARS-CoV2 S1-NTD is depicted in red.

The spike proteins found in the β-genus of *coronaviridae* share unique structural similarities with human Gal-3, suggesting a functional similarity. In fact, structural analysis of the NTD of the Spike protein sub-unit S1 in murine hepatitis virus (MHV) has shown a nearly identical topology to human Gal-3, with the only difference being two additional β-strands in one of the β-sheet layers of MHV S_1_ -NTD [24,25,26]. Additionally, a study of bovine coronavirus (BCoV) uncovered substantial overlap between the virus’s S_1_ -NTD receptor binding domain and the galactose-binding domain of human galectins; this finding is also clearly indicative of functional similarity [24,27] (Figure 3).

Several other studies have referred to the presence of “galectin fold” on the NTD of coronaviruses [13,28]. Indeed, the structures of Gal-3 and the S1-NTD of *betacoronaviridae* are so similar that it has been hypothesised that coronaviruses integrated a host galectin gene into their genome at some point in their evolution [25]. Given the prominent morphological, functional, and sequence similarities (12%) of the SARS-CoV2 NTD with human Gal-3, it is plausible that existing Gal-3 inhibitors possess dual-binding capabilities [13]. Such a mechanism, of course, would be promising in reducing viral entry to host cells.

**Figure 4:**
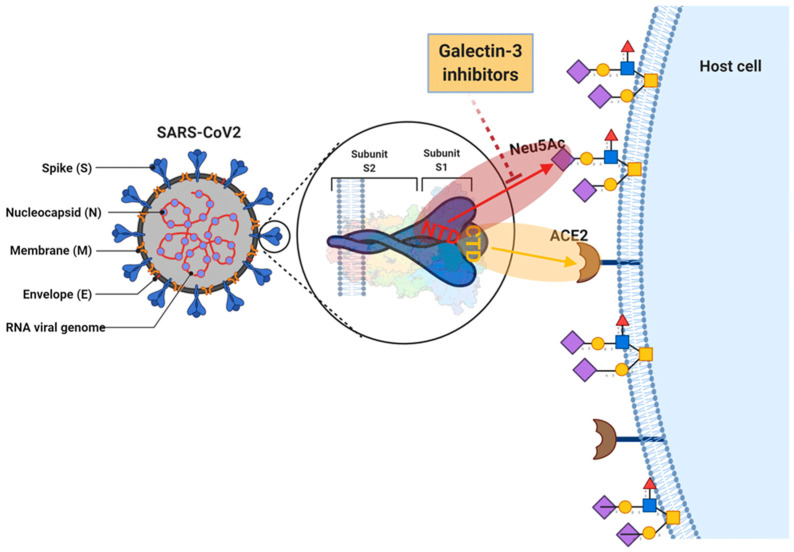
A dual attachment model for SARS-CoV2. This shows that a pocket in the NTD of SARS-CoV2 is capable of binding *N*-acetylneuraminic acid (Neu5Ac) [25] and strongly supports a dual attachment model for SARS-CoV2, where NTD-Neu5Ac interactions facilitate initial host cell recognition by the virus and stabilise its entry via ACE2 receptors. Adopted from [13] with permission.

Caniglia et al. have proposed a dual attachment model for SARS-CoV2 through Gal-3 and ACE2 (Figure 4). They suggested that a pocket in the NTD of SARS-CoV2 is capable of binding *N*-acetylneuraminic acid (Neu5Ac) [25]. As such, their study strongly supports a dual attachment model for SARS-CoV2, where NTD-Neu5Ac interactions facilitate initial host cell recognition by the virus and stabilise its entry via ACE2 receptors [13]. It is worth mentioning that HCoV-OC43 and HCoV-HKU1 use host sialosides as the only receptor in invading host cells. Furthermore, MERS-CoV operates as a dual binder to both human dipeptidyl peptidase-4 (DPP4) host protein receptor and host sialosides [10,11].

Gaetano et al. [29] drew the attention of the scientific community to S1-NTD as a putative target for designing small molecules to hinder the attachment of SARS-CoV-2 to host cells. They drew upon the recent literature identifying possible binding sites of S1-NTD and used a computational approach to detect the druggable cavities available in S1-NTD.

In general, there are four types of binding sites/domains in S1-NTD that need to be considered in docking calculations: (i) sialoside binding site, (ii) ganglioside binding domain, (iii) glycosylation sites (iv) a new cavity predicted by SiteMap [29] as particularly suited for setting up structure-based drug design approaches.

In summary, based on this evidence (i.e., considering the strict structural analogy and the stringent link between galectins and infection), it is reasonable to infer that binding to sugars could also involve the galectin fold. Indeed, it is widely known that galectins play a critical role in host–pathogen interactions, such as adhesion of pathogens to host cells and activation of host defence and adaptive immunity exposure to an antigen. Considering the high degree of structural and sequence similarity (12%) between S1-NTD and Gal-3, it is plausible that existing Gal-3 inhibitors, capable of modulating the interaction with a sugar, could bind the S1-NTD and could signify—another promising therapeutic application.

In what follows, with the aid of molecular docking studies, we shed light upon the structure, activity, and binding of 56 promising inhibitors interacting with Gal-3, considering all the binding sites of S1-NTD and S1-RBD in our docking calculations. Further details about the binding pockets are represented in the Materials and Methods section. As described below, we separately docked all the compounds on Gal-3 and Spike proteins, shortlisting the top scoring common compounds in terms of their performance as dual inhibitors. By broadening the knowledge base concerning dual Gal-3 and spike inhibitors, this work contributes substantively to the ongoing search for an efficacious therapy for COVID-19 infection.

## 2. Materials and Methods

### 2.1. Data Set Collection and Ligand Database Preparation

A set of 56 Gal-3 inhibitors was collected and utilised as a dataset for our docking studies. The chemical IDs of inhibitors along with their PDB codes and pKd values are listed in Table 1. K_d_ values are available for 14 of the compounds in the literature. We included the corresponding pKd values of them in Table 1. pKd was set equal to −1 for those inhibitors without experimental K_d_ values. For the last eight compounds, we could not find an identification code; therefore, henceforth we refer to them with “NEW1”, “NEW2”, “NEW3”, “NEW4”, “NEW5”, “NEW6”, “NEW7” and “NEW8”. Before the evaluation of the interactions between the Gal-3 structures and ligands, the database underwent a *Wash* procedure using the MOE (Molecular Operating Environment) software package in order to adjust the 3D structure and select the dominant protonation state of the molecules according to a physiological pH of 7.

### 2.2. Preparation of the Protein 

The atomic coordinates of galectin proteins were obtained from the Protein Data Bank (PDB) as 1KJR [42], 5E89 [30], 5ODY [32], 6EOL [38] and 6F2Q [43]. Gal-3 has an intrinsically disordered N-terminal domain, so no crystallographic information on Gal-3 beyond the lectin domain is available; consequently, we did not include N-terminal in our calculations. The missing hydrogens for heavy atoms were added using the tLEAP module of AMBER 14 with the AMBER14SB force field [44]. The protonation states of all ionizable residues were determined at pH = 7 using the MOE program [45]. Each protein model was solvated in a 12 Å box of TIP3P water. In order to bring the salt concentration to the physiological value of 0.15 M, Na^+^ and Cl^−^ ions were added. Minimization of the structure was carried out in two steps, using the steepest descent and conjugate gradient methods successively. At first, minimization was made in 2 ps on solvent atoms only, by restraining the protein–ligand complex. Next, minimization was run without the restraint over 10 ps. After minimization, the molecular dynamics (MD) simulations were carried out in three steps: heating, density equilibration, and production. At first, each solvated system was heated to 298 K for 50 ps, with weak restraints on all backbone atoms. Next, density equilibration was carried out for 50 ps of constant pressure equilibration at 298 K, with weak restraints. Finally, MD production runs were performed for all systems for 100 ns. The root-mean-square deviation (RMSD) of structures were found to reach a plateau after 30 ns. Clustering analysis of the last 70 ns of the generated MD trajectory was carried out using the Amber’s CPPTRAJ program [46] to identify representative conformations of each structure. Clustering was made via the hierarchical agglomerative approach using the RMSD of atoms in the colchicine binding site as a metric. An RMSD cutoff of 1.0 Å was set to differentiate the clusters. On the basis of the clustering analysis, two representative structures for 6F2Q (6F2Q_0 and 6F2Q_1) and one representative structure for 1KJR, 5E89, 5ODY and 6EOL were found, so in total six conformations were analysed. The docking was done on all of the six representative structures and the one with the highest docking score was selected.

The prepared structures of two possible conformation of Spike protein, namely the open state (PDB: 6VSB) and closed state (PDB: 6VXX), which are ready to be used as initial structures for docking simulations were adopted from CHARMM-GUI Archive of COVID-19 Proteins Library (http://www.charmmgui.org/?doc=archive&lib=covid19, accessed on 8 September 2021) [47,48,49].

### 2.3. Binding Sites Locater (Site Finder)

With regard to the NTD and RBD components of the Spike protein, we list the binding sites (i.e., the residues involved) that have been suggested in the literature to date in Table 2.

We also used the Site Finder function in MOE [54] to locate the possible binding sites in the NTD and RBD domains of the Spike protein. The PLB index, it should be noted, can be used to predict the ligand-binding sites of uncharacterised protein structures, as well as to identify the novel drug-binding sites of known drug targets [55]. All the sites we found using the MOE software had already been identified in the literature, so we continued with the binding site calculations mentioned in Table 2.

Milanetti et al. [50] presented iso-electron density mapping in support of the hypothesis that MERS-CoV bears a structural resemblance with SARS-COV-2. They also proposed a potential sialoside binding site (site 1) featuring three divergent loop regions. Behloul et al. [28], meanwhile, compared the structural characteristics of the S1-NTD from SARS-CoV-2 with BCoV and accordingly identified a binding pocket capable of binding sugars such as Neu5,9Ac2 (site 2). Baker et al. [51] aligned the sequences of coronavirus S proteins, focusing on the sialic binding protein, HCPV-OC42. They identified a potential sialic acid binding site, demonstrating its glycan-binding property using glyconanoparticles for the detection (site 3). Gaetano et al. [29] assessed the druggability of all the potential ligand-binding pockets within the S1-NTD using Schrodinger’s SiteMap tool [56]. 

Among the three sialoside binding pockets they proposed Baker et al. found that only site 3 exhibited a druggable property. (They refer to this binding site as P1 in their paper.) They also proposed an unexpected binding pocket, (site 4-P2), within S1-NTD [29]. This observation is in agreement with the recent experimental findings of Bangaru et al. [57], who put forward P2 as a relevant binding site for drug repurposing studies. It is worth mentioning that site 3 is a part of site 4, sharing the same binding residues. 

Sites 6 to 14 belong to the list of glycosylation binding sites proposed by Watanabe et al. [7]. Fantini et al. [52] identified a novel ganglioside-binding domain (GBD) using MD simulations, demonstrating a strong interaction between GM1 Ganglioside and S1-NTD (site 15). Carino et al. [53] proposed sites 16 to 20 in the RBD fragment of the Spike protein using the Fpocket server (https://bioserv.rpbs.univ-paris-diderot.fr/services/fpocket/, accessed on 8 September 2021). They proved that several triterpenoids (e.g., glycyrrhetinic and oleanolic acids) and natural bile acids and their semisynthetic derivatives can reduce RBD adhesion to the ACE2 consensus in vitro. Sites 21 to 22, meanwhile, are the Glycosylation binding sites proposed by Watanabe et al. [7].

To identify the most probable binding pockets of Gal-3, the *Site Finder* tool in MOE was used on all the representative proteins. Gal_Site1 was accordingly selected as the site with the highest propensity for ligand binding (PLB > 0.5) corresponding to the most known binding site for Gal-3.

### 2.4. Molecular Docking

Docking and scoring approaches provide the most promising route for drug design and discovery. The behaviour of small molecules in the binding sites of target proteins can be described by the identification of the correct poses of ligands in the binding pocket of a protein and to predict the affinity between them. Binding affinity is the strength of the binding interaction between a single biomolecule and its ligand partner and it is typically measured by the equilibrium dissociation constant (K_d_), the smaller the K_d_ value, the greater the binding affinity of the ligand for its target. Binding affinity is influenced by non-covalent intermolecular interactions such as hydrogen bonding, electrostatic interactions, hydrophobic and Van der Waals forces between the two molecules. In MOE software, receptor–ligand binding affinities are classified on the basis of a numerical value referred to as the “S-score”. Interactions of inhibitors with receptor proteins are predicted on the basis of the S-score, the higher the S-score (absolute value) the greater the interaction [58]. In this paper we used two methods of MOE software to calculate the S-score. First, we used triangle matcher procedure with the London dG scoring function to generate 30 poses and then used the rigid receptor procedure with the GBVI/WSA dG scoring function to refine them. The best five poses were kept at the end of refinement. During placement with the triangle matcher procedure, ligands are placed in the pocket by aligning triplets of atoms to triplets of alpha spheres, used by MOE to represent the binding site.

### 2.5. Regression Analysis

In Galectin-3, to study the correlation between the obtained docking score and experimental results, regression analysis was performed using a linear regression model. After having extracted the docking score and experimental pKd columns from the docking output database, the lowest-scoring pose of each compound with a known experimental pKd was selected. Regression analysis was performed using a custom-made script and the Scikit-Learn library in Python. Outlier detection procedure was performed using Density-Based Spatial Clustering of Applications with Noise (DBSCAN) with minimum five points required to form a dense region. Each compound was represented by a point with a docking score and experimental pKd coordinates. In this way, the outliner compounds (points far from the main distribution) can be identified and discarded for the regression analysis. Therefore, the linear regression model was applied to the data set. The results of the linear regression were evaluated through the R^2^ parameter.

### 2.6. Protein–Ligand Interaction Fingerprint (PLIF)

The Protein–Ligand Interaction Fingerprint (PLIF) descriptors implemented in MOE were used to summarise the interactions between ligands and proteins using a fingerprint scheme. Interactions are classified as hydrogen bonds, ionic interactions, and surface contacts and converted into a fingerprint scheme which is demonstrative of a given database of protein-ligand complexes.

### 2.7. Toxicity Risk

Various toxicity- and ADMET-related properties of the investigated compounds were predicted in silico using the toxicity module of ADMET Prediction™ (version 9.5, Simulation Plus, Lancaster, CA, USA) software, where a broad range of toxicities are covered including cardiac, hepatotoxicity, endocrine, carcinogenicity and sensitivity [50,59] ADMET Risk and ADMET Code for toxic liability are TOX_Risk and TOX_Code, respectively. The TOX_Risk model consists of seven rules, including one based on TOX_MUT_Risk. Each has an associated weight of one. A list of abbreviations used here is given below (Table 3):

TOX Risk rule codes for toxicity are as follows: hERG = hERG inhibition, rat = acute rat toxicity, Xr = carcinogenicity in rat, Xm = carcinogenicity in mice, HEPX = hepatotoxicity, MUT = Ames positive. The possible value range for TOX_MUT_Risk is 0–11 and it is 0–7 for TOX_Risk

## 3. Results

We docked the database of compounds to the Spike (PDBID: 6vsb- open and 6vxx- closed) and Gal-3 (PDBID: 6F2Q_0, 6F2Q_1, 1KJR, 5ODY, 6EOL and 5E89) proteins. The details of the results and discussions will be presented in the following sections.

### 3.1. Regression Analysis on Galectin-3

**Figure 5:**
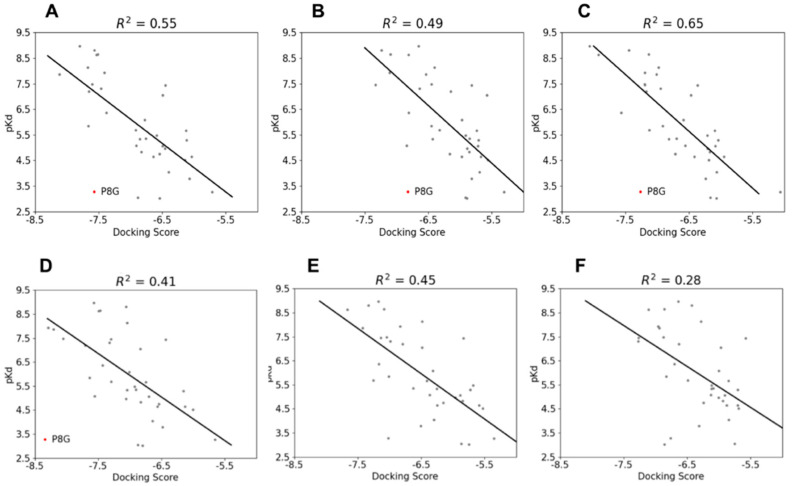
Regression analysis of blind docking results. Linear regression plots of (**A**) 6F2Q_0, (**B**) 6F2Q_1, (**C**) 1KJR, (**D**) 5ODY, (**E**) 6EOL and (**F**) 5E89 illustrate the blind docking results. The outliers detected during clustering are highlighted in red and their names are shown in the image.

As experimental pKd values were only available for the Galectin-3 protein in the literature, we only performed regression analysis to evaluate the correlation between the obtained docking scores and experimental pKd values for six different protein structures available in Protein Data Bank. In four structures out of six, compound P8G was recognised as an outlier of the data distribution, meaning that the docking experiment was not able to predict properly its binding affinity. The best correlation between computational and experimental results was found for the 1KJR structure (R^2^ = 0.65). Regression plots and the corresponding R^2^ score are shown in Figure 5.

Based on the regression analysis, we reported the docking scores for the database encompassing the PDB:1KJR structure of Gal-3.

### 3.2. Molecular Docking Calculations

We docked the Galectin-3 inhibitor database on Spike (open and closed conformations) and Galectin-3 proteins. All docking scores and binding sites are reported in Table 4. In the following sections, we discuss the top eight Gal-3 (score > 7.5) and the top eight Spike protein inhibitors, as well as the common inhibitors within the 8 top ranked inhibitors. 

The top inhibitors for Gal-3 and Spike proteins were found to be (A6J, NEW1, NEW4, NEW6, 8VT, GMK, NEW8, TD2) and (P8G, A6J, GMK, 8VT, NEW7, NEW8, TD2, NEW5), respectively. The common best inhibitors for both Spike and Gal-3 (A6J, 8VT, GMK, NEW8, TD2) are signified with an asterisk (*) in Table 4. 

**Figure 6:**
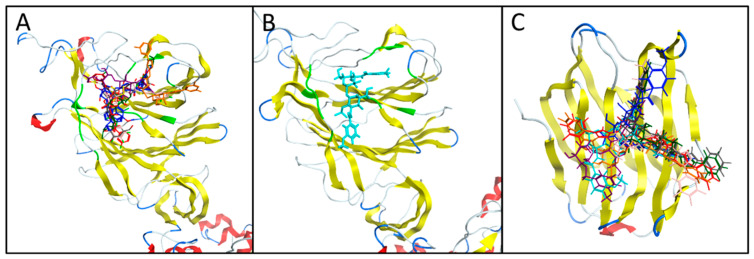
Binding poses of (**A**) A6J (dark-blue), NEW8 (orange), 8VT (purple) and TD2 (red), NEW7 (pink), NEW5 (dark green) and p8G (dark grey) on (**A**) S1-NTD domain open conformation, (**B**) GMK (cyan) on S1-NTD domain closed conformation, and (**C**) binding poses of on Gal-3 with the same colour codes mentioned in (**A**,**B**) parts except NEW6 (dark gray), NEW4 (pink) and NEW1(dark green). The site 4 (sialoside site) residues are highlighted in light green.

All the compounds considered, with the exception of 8VT, were found to bind to site 4 of S1-NTD, which is a sialoside binding site. Site 4 was found to have residues in common with Baker et al.’s site 3, to which it is adjacent (Figure 6A). Meanwhile, 8VT was found to bind to site 5. Site 3, site 4 and site 5, it should be noted, are all sialoside binding sites featuring at least one glycosylation site (Figure 6B). It was also found that GMK binds to site 4 of the closed conformation of the Spike protein (Figure 6C). 

Interestingly, the compounds with higher affinity for NTD are in the top of list of the compounds with higher scores (affinity) for the RBD domain as well. As for Gal-3, all the compounds were found to bind to the galactose binding site (Gal_site1). 

The binding poses of the all the compounds with high affinity for Spike and Gal-3 are shown in Figure 6. In the Spike protein, the inhibitors were found to bind to site4 (sialoside site) with the exception of NEW8 (orange) binding to site 5 (another sialoside site), seven of them in an open conformation (A6J, GMK, NEW8, 8VT, NEW7, NEW5 and TD2) and one of them (GMK) in a closed conformation. All the Gal-3 inhibitors were found to bind to the known galactose binding site in Gal-3 (Gal_site1). 

In what follows, two parallel analyses will be considered. First, the top eight inhibitors (i.e., those with the highest S-scores) for Gal-3 protein (A6J, NEW1, NEW4, NEW6, 8VT, GMK, NEW8, TD2) and their interactions will be discussed (Section 3.3). Then, the top eight inhibitors (i.e., those with the highest S-scores) for Spike protein and their interactions will be analyzed (P8G, A6J, GMK, 8VT, NEW7, NEW8, TD2, NEW5) (Section 3.4). Finally, we provide an overall discussion of dual inhibitors in Section 3.5.

### 3.3. Galectin-3 Protein Interactions with Top Ranked Inhibitors

**Figure 7:**
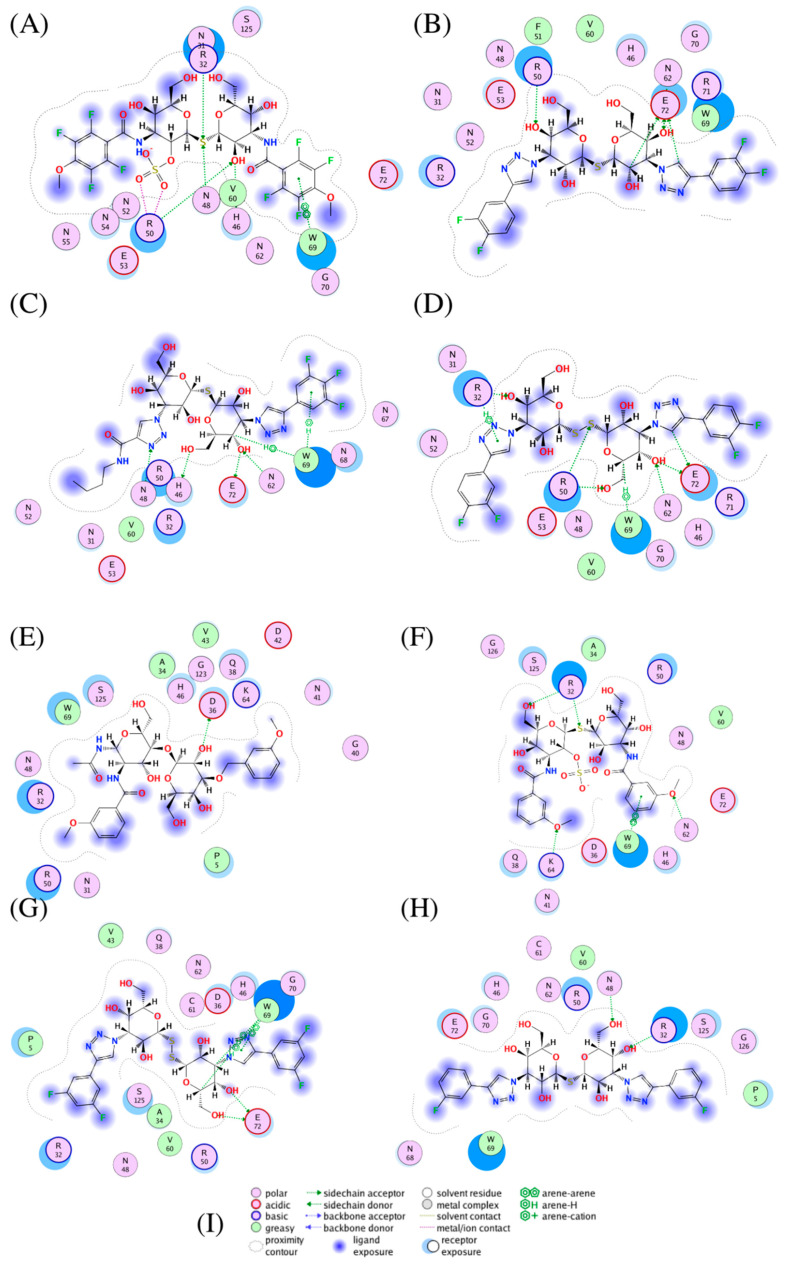
Ligand interaction plots of compounds selected for Gal-3 inhibition. (**A**) A6J (**B**) NEW1, (**C**) NEW4, (**D**) NEW6 (**E**) 8VT (**F**) GMK (**G**) NEW8 and (**H**) TD2. A graphical key (**I**) is included to help interpret the 2-D part of the ligand interactions panel.

A6J obtained the lowest docking score, sharing similar structures with GMK (which obtained the sixth-lowest score). Despite having similar structures, these two compounds shared only two interactions—an arene–arene interaction with W69, common among compounds with high experimental affinity, and a sidechain acceptor interaction with R32, in particular through the sulphur atom linking the two saccharides (Figure 7A–F). A6J also developed interactions with R50 (ionic and sidechain acceptor) and H46 (sidechain donor). It should be noted that each of these two compounds is characterised by an amide—rather than a triazole—structure linking the aromatic rings to the thiodigalactosides. Meanwhile, one of the sulphide groups exhibited the ability to develop strong interactions with R50. The main differences between these two compounds were found to be in the aromatic rings, where A6J is fluorinated and shows the methoxy group in a different position.

NEW 1 was the second-best-performing compound according to our docking score, and was the element in our database with the highest experimental affinity for Gal-3. NEW1 has a molecular structure very similar to that of NEW6, which has two sulphur atoms—rather than one—bridging the saccharide units. Both NEW1 and NEW6 were found to interact with R50 (sidechain acceptor), N62 (sidechain acceptor) and E72 (sidechain donor), while only NEW6 interacted with R32 (arene and sidechain donor) and W69 (arene) (Figure 7B–D).

NEW 4 was found to differ from the other selected compounds because it has only one aromatic ring; indeed, the second hydrophobic group is represented by a butyl functionality. As for NEW1, it exhibited interactions with N62 (sidechain acceptor), W69 (arene) and E72 (sidechain donor) (Figure 7C), as well as with H46 (sidechain donor) and N48 (sidechain acceptor).

The NEW8 compound was found to exhibit several arene interactions with W69 and two sidechain donor interactions with E72 (Figure 7G). The 8VT compound, meanwhile, was characterised by a sidechain donor interaction with D36 (Figure 7E).

TD2 has been widely cited in the literature as a Gal-3 inhibitor. In fact, TD2 is currently undergoing clinical trials with promising results in reducing symptoms of pulmonary fibrosis [60]. With a half-maximal inhibitory concentration (IC_50_) of 361 nM, it has been shown to be well-tolerated in humans when inhaled [61]. In the obtained pose, TD2 interacts with R32 and N48 with sidechain acceptor interactions (Figure 7H). Moreover, TD2 (also referred to as TD-139) has been shown to have a promising interaction with the RBD of Spike protein, and has been proposed as a potential inhibitor of RBD-ACE2 binding [62].

### 3.4. Spike Protein Interactions with Top Ranked Inhibitors

The eight compounds showing the highest S-scores in relation to the Spike protein are similar in structural characterization. The majority have two saccharide units, linked by at least one sulphur atom, and hydrophobic groups at the sides.

**Figure 8:**
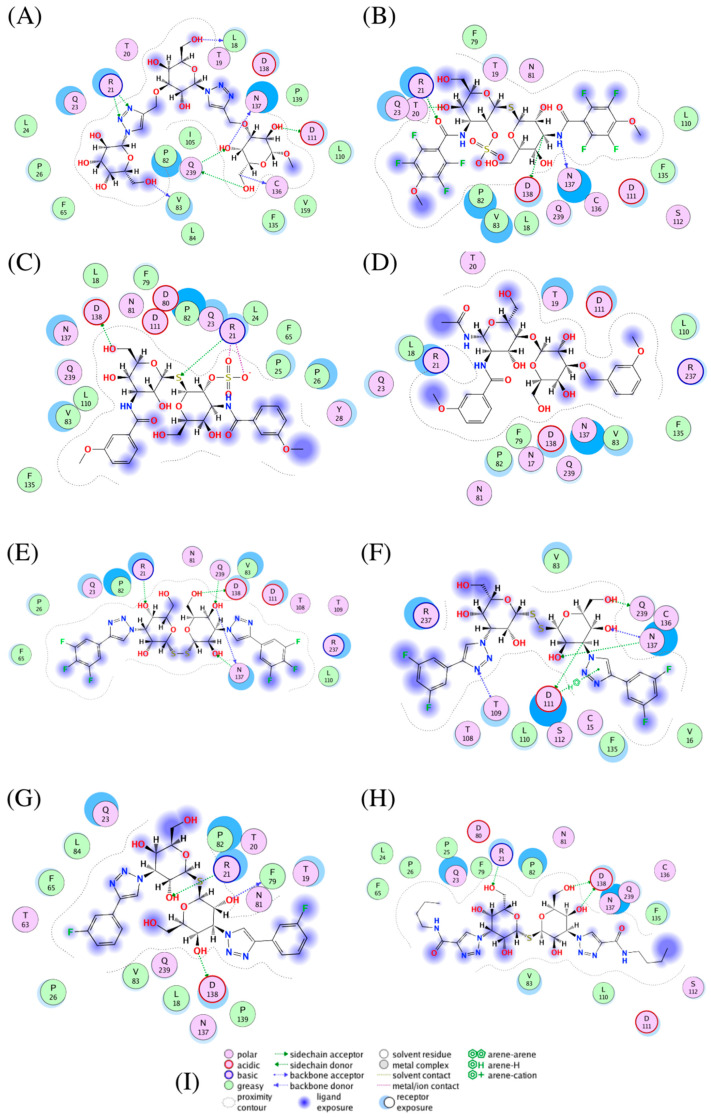
Interaction plots of five top Spike protein inhibitors. (**A**) P8G, (**B**) A6J, (**C**) GMK, (**D**) 8VT, (**E**) NEW7, (**F**) NEW8, (**G**) TD2 and (**H**) NEW5. A graphical key (**I**) is included to help interpret the 2-D part of the ligand interactions panel.

The highest S-score in relation to the Spike protein was that obtained by P8G—the only one characterised by three saccharide units, all involved in interactions with the protein. P8G’s top-scored pose exhibited two sidechain acceptor interactions between the two nitrogen atoms—engaged in the double bond of the triazole—and the R21 residue. It shared sidechain acceptor and donor interactions with Q239. It also presented a sidechain donor interaction with D111, as well as three backbone bond donors with V83, L18 and the C136 residues (Figure 8A).

A6J, which was found to have the second-highest docking score, interacted with R21 in a sidechain acceptor interaction (Figure 8B). Moreover, it had a backbone donor bond with the residue N137 and a sidechain donor interaction with D138. Finally, solvent contact was detected between T20 and one of the hydroxyl groups of the thiodigalactoside.

Despite their structural similarities, GMK exhibited different interactions from A6J (Figure 8C). Indeed, it had two ion contacts between its sulphate ion and the R21 residue. These kinds of interactions have not been previously observed with respect to the A6J compound, probably due to the interior position of the sulphate ion. It should be noted that the same R21 residue interacts with A6J, once again with a sidechain acceptor interaction with the sulphate belonging to the thiodigalactoside. Finally, a sidechain donor interaction with the N138 residue was detected. The lower S-score for GMK may be attributable to the absence of fluorine atoms on the external aromatic rings and the methoxy group being in a different position. Further, 8VT, meanwhile, had some of the characteristic groups that are recurrent in these compounds and exhibited an arene interaction with R21 (Figure 8D).

NEW7 and NEW8 are characterised by two sulphur atoms bridging the saccharide units, and they differ only in the number of fluorine atoms in the aromatic rings. NEW7 interacted with Spike NTD via three sidechain acceptor interactions (with N21, N137, and Q239). It also presented a sidechain donor interaction with D138 and a backbone donor bound with N137. NEW8, on the other hand, was characterised by two sidechain donor interactions (with N137 and T109) and a backbone donor bound with N137. Moreover, an arene interaction with D111 and sidechain acceptor interactions with D111 and Q239 were observed (Figure 8E,F).

TD2, which presents only one sulphur atom, was characterised by a sidechain donor interaction with R21, a backbone acceptor interaction with F79, and a sidechain acceptor interaction with D138. The same interactions with R21 and D138 were observed in the case of NEW5, which is characterised by the absence of the two aromatic rings, replaced by butyl groups (Figure 8G).

### 3.5. Protein Ligand Interaction Fingerprints (PLIF) Analysis

**Figure 9:**
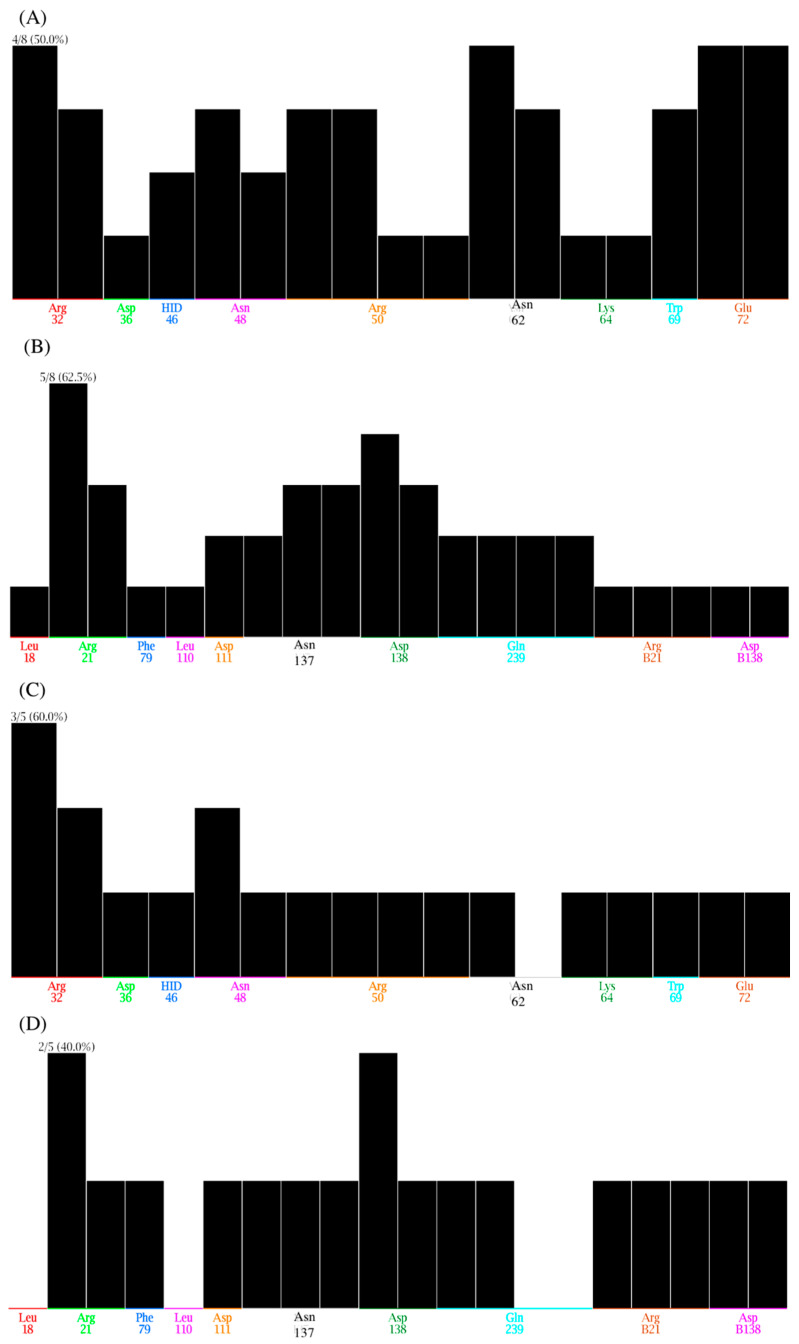
Population histogram of PLIF results displaying the number of ligands (*y*-axis) with which each residue (plotted in the *x*-axis) interacts. Population display of (**A**) top-scoring inhibitors in Gal-3 protein, (**B**) top-scoring inhibitors in Spike protein, (**C**) dual inhibitors in Gal-3 protein and (**D**) dual inhibitors in Spike protein.

The PLIF descriptors implemented in MOE were used to summarise the interactions between ligands and proteins using a fingerprint scheme. In this method, the interactions are first classified as either hydrogen bonds, ionic interactions, or surface contacts, and are then converted into a fingerprint scheme demonstrative of a given database of protein–ligand complexes. The population histogram (Figure 9) shows the number of ligands (*y*-axis) with which each residue (*x*-axis) was found to interact in our study. In the figure, each bar corresponds to one type of interaction for a given residue. So, for example, N111 has three types of interactions with most of the compounds.

In the case of Gal-3, the contacts are R32, D36, H46, N48, R50, N62, K64, W69, and E72. In the case of spike, the contacts are L18. R21, F79, L110, D111, N137, D138, and Q239. RB21 (ArgB21) and D138 (AspB138) belong to the Spike closed conformation. Those amino acids that have a counterpart in both spike and Gal-3 are R, D, and N. Gal-3 and spike dual inhibitors have the same contacts as the top-ranked inhibitors with the exception that L18 and L100 contacts are missing in Gal-3 protein.

### 3.6. Toxicity Analysis

For any compound to be considered as a potential drug candidate, it should have acceptable pharmacokinetic and pharmacodynamic profiles, as well as a high safety margin with lower chances of toxicity and adverse side effects. The different toxicity and ADMET-related properties of all the investigated compounds were predicted in silico using the toxicity module of the ADMET Predictor™ (version 9.5, Simulation Plus, Lancaster, CA, USA) software covering a large range of toxicities including cardiac, hepatotoxicity, endocrine, carcinogenicity and sensitivity (see Table 5) [59,63].

According to the toxicity risk evaluation, A6J, NEW8 compounds from our selected list of compounds (TD2, NEW8, AJ6, 8VT and GMK) show a toxicity risk value higher or equal to 2. In conclusion, considering only the toxicity risk, TD2, 8VT and GMK will be considered as potential candidate dual inhibitors for further in vitro and in vivo experiments.

### 3.7. Coronavirus Variants

As viruses replicate, small copying errors known as mutations naturally arise in their genomes. A lineage of coronaviruses will typically accumulate one or two random mutations each month. Some mutations have no effect on the coronavirus proteins made by the infected cell. Other mutations might alter a protein’s shape by changing or deleting one of its amino acids, the building blocks that link together to form the protein. Through the process of natural selection, neutral or slightly beneficial mutations may be passed down from generation to generation, while harmful mutations are more likely to die out [64]. After the outbreak of Covid-19, multiple SARS variants of the virus have been circulating globally. The most virulent ones emerged in the United Kingdom (known as B.1.1.7, Alpha), in South Africa (known as 20H/501Y.V2 or B.1.351, Beta), Brazil (known as P.1, Gamma) and in India (known as B.1.617.2, Delta). These viral mutations could have multiple consequences such as the ability to spread more rapidly in people, the ability to evade detection by specific viral diagnostic tests, the possibility to cause either milder or more severe disease in people, the decreased susceptibility to therapeutic agents such as monoclonal antibodies and the capability to evade natural or vaccine-induced immunity. Regarding the South African and Brazilian variants, the mutation is connected to the receptor binding domain (RBD). The RBD domain plays an important role in the diffusion of the virus. In fact it is proved that SARS-CoV-2 spreads faster than the previous SARS-CoV because of the mutations in the receptor binding domain (RBD) [65]. We summarised amino acid mutations of SARS-CoV-2 Alpha, Beta, Gamma and Delta variants of SARS-CoV-2 with a focus on Spike protein in Table 6. 

The fingerprint amino acids of the selected inhibitors to Spike protein were found to be L18, R21, F79, L110, D111, N137, D138, and Q239. The only mutation with the potential to have an adverse effect on the compounds under study is L18F in the Beta variant, which has a very low frequency in the fingerprint population histogram. Furthermore, the mutation of N501 [66], which is the most questioned mutation, does not affect this study. The Alpha, Beta and Gamma variants, it should be noted, have the same mutation (N501Y) in common which, in the SARS-CoV-2 S1 RBD, has been shown to possess increased affinity for ACE2 and making coronaviruses more contagious. Due to the residues of the binding site of our inhibitors do not have the mutation sites in common, we do not expect that the given variant will have a significant bearing on the binding of the selected compounds.

## 4. Discussion

Identifying potential drugs can be challenging due to the high cost of experimental investigation. Alternatively, protein–ligand docking is a popular and powerful computational technique to acquire the necessary information concerning drug and target interactions. In a recent study employing this technique, Sethi et al. [62] tested 330 galectin inhibitors against SARS-CoV-2 spike (S) protein. This study led to the in vitro testing of the ligand TD-139. Comparing the set of ligands that the authors elected to analyse with our own database, though, we found only a few compounds in common. Moreover, our study looks both at NTD domain binding sites and at RBD domain binding sites, while Sethi et al. focused solely on RBD. TD139, it should be noted, is a well-known galectin inhibitor and its interaction with the galectin fold has been demonstrated in previous studies. Moreover, in addition to the galectin fold in RBD, there are three glycosylation sites in the galectin fold of NTD (N122, N149, and N165). Therefore, the possibility of interaction between the glycans on the NTD and Gal-3 on the host warrants further consideration, and docking calculations on NTD and RBD have yet to be performed. In addition, inhibiting the NTD domain can have an allosteric effect on the binding of RBD to ACE2, so the NTD domain and its binding sites needs to be considered in docking calculations. In this context, interestingly, Olotu et al. employed all atom-MD simulation methods to investigate the allosteric effect of two high-affinity binders of S1-NTD on conformational alterations across the protein structures, including the RBD where hACE2 interactions occur. They observed that allosteric binding of both compounds disturbed the prefusion S-protein conformations, resulting in ACE2 displacement from the RBD. 

The top inhibitors for Gal-3 and Spike proteins were found to be (A6J, NEW1, NEW4, NEW6, 8VT, GMK, NEW8, TD2) and (P8G, A6J, GMK, 8VT, NEW7, NEW8, TD2, NEW5), respectively. 

In Gal-3 (Section 3.3), sidechain donor interactions with E72 and sidechain acceptor interactions with R32 and N62 were found to be present in half of the compounds considered. Hence, these residues may be crucial in the binding of Gal-3 to inhibitors. Less common were the arene interactions with W69 and sidechain acceptor interactions with R50 and N48, as they were found to occur in only three of the eight ligands. In Spike (Section 3.4), of the eight compounds under study, six were found to feature sidechain acceptor interactions with R21 while five featured sidechain donor interactions with D138. These residues may be crucial in high affinity interactions of Spike NTD with possible inhibitors. Interestingly, an arginine was found to be involved in the interactions between Gal-3 and its inhibitors. Less common was the backbone donor interaction with N137, which occurred in only three of the eight ligands. At the same time, our results show that potential inhibitors of Gal-3 share a similar structure, and that the introduction of a disulphide linking the two glucopyranose is able to further increase the affinity for this protein.

With the aim of finding potential dual inhibitors of Galectin-3 and Spike protein, the molecular docking results on the two proteins were compared. The common best inhibitors for both Spike and Gal-3 are (A6J, 8VT, GMK, NEW8, TD2). All of them are characterised by two saccharide units and aromatic rings with hydrophilic functionalities. The saccharide units are bridged by one sulphur atom, with the exceptions of NEW8, which has two sulphurs atoms, and 8VT, in which there is an oxygen atom. Moreover, A6J and GMK have a sulphide group capable of forming strong ionic interactions. Common residues involved in the interactions of Galectin-3 with these potential dual inhibitors are R32 and W69, R21 and D138 in case of Spike protein.

All Spike inhibitors bind to a sialoside binding pocket in NTD and they all prefer open conformation of Spike protein, except the GMK compound. These inhibitors can disrupt a dual attachment model for SARS-CoV2, where NTD-Neu5Ac interactions facilitate initial host cell recognition by the virus and stabilise its entry via ACE2 receptors. 

We found the important residues taking part in binding of the selected inhibitors in Section 3.5 and we compared them with amino acid mutations of SARS-CoV-2 Alpha, Beta, Gamma and Delta variants of SARS-CoV-2 with a focus on Spike protein that was summarised in Section 3.7. None of the residues of the binding site of our inhibitors have a mutation site in common in the variants except L18F in the Beta variant, leaving our compounds unaffected by the variants so far. 

We also filtered two dual compounds from our list (A6J, NEW8) after ADMET toxicity assessment in Section 3.6. The remaining compounds (TD2, 8VT and GMK) can serve as templates for developing leads and should be subjected to structural optimisation to generate clinically useful structures. In general, the optimization of natural lead structures may involve efforts on three different fronts: to enhance drug efficacy, to optimise absorption, distribution, metabolism, excretion and toxicity (ADMET) profiles and to improve chemical accessibility. In recent years, more attention has been devoted to optimization of ADME properties and reduction of toxicity, since their structural complexity might confer unfavourable effects on their pharmacokinetic properties, such as solubility, cellular permeability, and chemical or metabolic stability. Eventually, rational drug design such as design of new derivatives may overcome this bottleneck and eventually lead to the therapeutic application of these drugs.

## 5. Conclusions

The main idea of the study described herein was to find a dual inhibitor of both Gal-3 and the SARS-CoV2 S_1_- NTD that could reduce the release of cytokines and prevent viral attachment to host cells. We performed a docking and a filtering procedure to identify and rank potential dual-inhibitors from a database of 56 compounds, 37 of which have known experimental affinity with Gal-3. 

According to our docking simulations on Spike and Gal-3, we found five potential dual inhibitors, namely A6J, 8VT, GMK, NEW8 and TD2. We demonstrated the importance of residues R32, D36, H46, N48, R50, N62, K64, W69 and E72 for Gal-3 and of R32, D36, H46, N48, R50, N62, K64, W69 and E72 for Spike-NTD. Gal-3 and spike dual inhibitors have the same contacts as the top-ranked inhibitors with the exception that L18 and L100 contacts are missing in Gal-3 protein. 

We also evaluated the ADMET toxicity risk of these compounds using machine learning methods. The result of this study is a short-list of four candidate molecules (TD2, 8VT, NEW6 and GMK) for in vitro and in vivo testing to evaluate their inhibitory capabilities and to verify their potential to reduce viral entry and mitigate the release of TNF-α, IL-1β, and IL-6 from infected cells. 

It is worth mentioning that TD2 is currently undergoing a clinical trial and has shown no side effects in the trials carried out to date. Further studies can be carried out to analyse in detail the correlation between structure and activity of these compounds, perhaps expanding the ligand database; this information could be useful in informing the search for similar compounds. Moreover, these molecules are the first drug candidates to be identified that are proposed to reduce both viral entry and the inflammatory responses associated with infection. 

We also analysed the effect of new variants of SRAS-Cov-2 on our calculations. It is noteworthy that our results were not found to be affected significantly by these mutations. The mutation of N501, which is the most concerning one, does not affect the binding site of the binding compounds. The only mutation which could have adverse effects on the affinity of the compounds is L18F in the Beta variant, but we expect its impact to be marginal.

Finally, it is important to briefly outline the limitations of the in silico methods used in this study. These are predictive methods with inherent inaccuracies due to several sources of error. The first limitation is their reliability of the crystal structures of the protein targets which are obtained under specific experimental conditions, not precisely those occurring physiologically. These crystallographic structures have limited resolutions, typically on the order of 2 angstroms. Next, docking of the chemical structures (Figure S1) of the ligands is an algorithmic process with built-in limitations that are mainly due to the protein flexibility which is a computational challenge usually partially resolved by using several most dominant conformations obtained from molecular dynamics simulations. This is also dependent on the length of the simulation, which are becoming more realistic, but still not comparable to biological times of protein dynamics. Finally, the force fields used in obtaining free energy estimates of the ligand-protein binding do not provide accurate absolute values but, instead, are reasonably reliable in ranking the ligands’ affinities for the target protein. For this reason, a rank order for a panel of ligands is the most reliable predictor provided by computational methods. With partial experimental data one can calibrate the free energy values and refine the computational model.

In conclusion, following an in vitro validation of our in silico predictions, the use of Gal-3 inhibitors could lead to a modulation of the host immune response and consequently the incidence of CSS, a reduction of the incidence of post-infection pulmonary fibrosis, and the prevention of viral entry, exploiting dual targets for both Gal-3 and spike protein.

## Figures and Tables

**Table 1:** Ligand database.

#	Name	PDB	pKd for Gal-3	Reference
1	5KS	5E8A	7.469	[30]
2	5KT	5E88	7.187	[30]
3	5SY	5EXO	−1	-
4	DQT	3T1M	3.041	[31]
5	MQT	3T1L	3.260	[31]
6	9Q5	5OAX	7.921	[32]
7	9SK	5ODY	8.125	[32]
8	BEK	-	−1	-
9	EGZ	6G0V	3.780	[33]
10	8VT	5NFB	6.357	[34]
11	8VW	5NF9	5.328	[34]
12	P8J	6Q17	3.012	[35]
13	H5T	6I75	4.638	[36]
14	H5Z	6I74	5.469	[36]
15	H5Q	6I77	4.959	[36]
16	H5N	6I76	5.071	[36]
17	H5H	6I78	4.745	[36]
18	P8G	6Q0Q	3.272	[35]
19	J5Q	6QLN	5.658	[32]
20	J3Q	6QLP	5.284	[32]
21	J5E	6QLO	5.056	[37]
22	J4N	6QLR	4.824	[37]
23	J4E	6QLQ	4.509	[37]
24	J5W	6QLT	4.036	[37]
25	HRK	6QLS	5.347	[37]
26	J62	6QLU	4.638	[37]
27	UNU	-	−1	-
28	THR	-	−1	-
29	E61	-	−1	-
30	BKH	6EOL	7.428	[14]
31	BKK	6EOG	7.310	[38]
32	TD2	5H9P	7.854	[39]
33	TGZ	5H9R	6.076	[39]
34	J1E	6QGE	5.678	[40]
35	KP8	6RZI	−1	-
36	KOZ	6RZK	−1	-
37	KPB	6RZJ	−1	-
38	KON	6RZM	−1	-
39	KOE	6RZL	−1	-
40	J0T	6QGF	5.839	[40]
41	A6J	4BM8	−1	-
42	GMK	4BLI	−1	-
43	70B	4BLJ	7.301	[41]
44	KOW	6RZG	−1	-
45	KP5	6RZF	−1	-
46	KOQ	6RZH	−1	-
47	GCU	-	−1	-
48	NAG	-	−1	-
49	NEW1	-	8.959	[32]
50	NEW2	-	8.638	[32]
51	NEW3	-	8.796	[32]
52	NEW4	-	8.620	[32]
53	NEW5	-	7.446	[14]
54	NEW6	-	-	-
55	NEW7	-	-	-
56	NEW8	-	-	-

**Table 2:** Binding sites of Spike NTD and RBD obtained from the literature.

Binding Sites	Reference	Binding Site Type [Reference]	Residues	RBD/NTD Open/Closed
Site1	Milanetti et al. [50]	Sialoside	L18-Q23, H66-T78, and G252-S254	NTD
Site2	Behloul et al. [28]	Sialoside	E154, F157, Y160 and the so-called stabilizing loop (N122-N125)	NTD
Site3	Baker et al. [51]	Sialoside	(R21, Q23, L24, H69, F79, P82, and R246)	NTD
Site4 (P1)	Gaetano et al. [29]	Sialoside	R21, T22, Q23, L24, P26, R78, P82, V83, L110, F135, C136, N137, and R237	NTD
Site5 (P2)	Gaetano et al. [29]	Sialoside	F92, S94, E96, K97, S98, R102, N121, V126, I128, M177, D178, K182, N188, R190, F192, I203, L226, V227, and L229.	NTD
Site6-14	Watanabe et al. [7]	glycosylation	N122, N149,N165, N17,N61, N74, N234, N282	NTD
Site15	Fantini et al. [52]	ganglioside	Domain (111–158)- core Q-134 to D-138	NTD
Site16	Carino et al. [53]	-	F342 N343 A343 T345 R346–W436 N437 S438–L441 D442 S443–G446–N448–Y451 L452	RBD
Site17	Carino et al. [53]	-	S375–G404 D405–V502 G503–Q506–Y508	RBD
Site18	Carino et al. [53]	-	E340 V341–F347 A348–N354 R355 K356–S399 F400 V401–V512	RBD
Site19	Carino et al. [53]	-	F374–N388–Y495 G496 F497	RBD
Site20	Carino et al. [53]	-	T376 F377 K378 C379 Y380–V407 R408 –I410–V433 I444 A445	RBD
Site 21-22	Watanabe et al. [7]	Glycosylation	N331-N334	RBD

**Table 3:** Abbreviations.

hERG liability	hERG: TOX_hERG > 6
acute toxicity in rats	ra: TOX_RAT < 300
carcinogenicity in chronic rat studies	Xr: Rat_TD50 < 4
carcinogenicity in chronic mouse studies	Xm: Mouse_TD50 < 25
hepatotoxicity	Hp: (TOX_AlkPhos = Toxic OR TOX_GGT = Toxic OR TOX_LDH = Toxic) AND (TOX_SGOT = Toxic OR TOX_SGPT = Toxic)
SGOT and SGPT elevation	SG: TOX_SGOT = Toxic AND TOX_SGPT = Toxic
Mu	TOX_MUT_Risk > 2

**Table 4:** Selected top-ranked inhibitors for Galectin-3 and Spike proteins. Dual inhibitors are indicated by an asterisk (*).

Galectin-3			Spike		
Name	Score	Binding Site	Name	Score	Binding Site
A6J *	−8.125	Gal-site1	P8G	−9.151	open-NTD-site4
NEW1	−8.059	Gal-site1	A6J *	−8.439	open-NTD-site4
NEW4	−7.919	Gal-site1	GMK *	−8.209	close-NTD-site4
NEW6	−7.629	Gal-site1	8VT *	−8.168	open-NTD-site4
8VT *	−7.561	Gal-site1	NEW7	−8.164	open-NTD-site4
GMK *	−7.557	Gal-site1	NEW8 *	−8.093	open-NTD-site5-P2
NEW8 *	−7.554	Gal-site1	TD2 *	−8.091	open-NTD-site4
TD2 *	−7.551	Gal-site1	NEW5	−8.057	open-NTD-site4
NEW2	−7.314	Gal-site1	NEW2	−8.013	open-NTD-site4
P8G	−7.260	Gal-site1	9SK	−7.969	close-NTD-site14 and site 5-P2
5KS	−7.191	Gal-site1	9Q5	−7.95	open-RBD-site22
9Q5	−7.191	Gal-site1	NEW3	−7.934	open-NTD-site4
NEW5	−7.190	Gal-site1	8VW	−7.895	close-NTD-site10
5KT	−7.168	Gal-site1	NEW4	−7.864	open-NTD-site4
NEW3	−7.129	Gal-site1	70B	−7.857	open-NTD-site10
J1E	−7.121	Gal-site1	5KS	−7.817	open-RBD-site21
9SK	−6.974	Gal-site1	NEW6	−7.685	close-NTD-site4
70B	−6.943	Gal-site1	5KT	−7.673	open-NTD-site10
NEW7	−6.920	Gal-site1	J1E	−7.621	close-NTD-site9 and site 4
J0T	−6.910	Gal-site1	NEW1	−7.544	open-NTD-site10
KOW	−6.853	Gal-site1	J0T	−7.355	close-NTD-site9
KON	−6.852	Gal-site1	P8J	−7.35	open-NTD-site4
KOQ	−6.826	Gal-site1	E61	−7.263	open-NTD-site4
8VW	−6.813	Gal-site1	H5Q	−7.25	open-NTD-site10
KP5	−6.714	Gal-site1	KOW	−7.231	open-NTD-site10
H5H	−6.712	Gal-site1	KP5	−7.194	open-NTD-site10
HRK	−6.692	Gal-site1	HRK	−7.145	open-NTD-site3
KOZ	−6.629	Gal-site1	TGZ	−7.141	close-NTD-site14
KPB	−6.616	Gal-site1	H5H	−7.122	open-NTD-site10
TGZ	−6.584	Gal-site1	BKH	−7.089	open-NTD-site3
H5N	−6.555	Gal-site1	J5W	−7.083	close-NTD-site10
BKK	−6.465	Gal-site1	KOQ	−7.056	open-NTD-site10
E61	−6.428	Gal-site1	J62	−6.992	open-NTD-site10
H5T	−6.412	Gal-site1	J4E	−6.987	close-NTDsite10
KP8	−6.397	Gal-site1	KOE	−6.978	open-NTD-site3
BKH	−6.362	Gal-site1	KON	−6.954	open-NTD-site10
5SY	−6.268	Gal-site1	J4N	−6.948	close-NTD-site14
EGZ	−6.235	Gal-site1	H5Z	−6.946	open-NTD-site10
H5Z	−6.208	Gal-site1	EGZ	−6.945	open-NTD-site9
H5Q	−6.206	Gal-site1	J5Q	−6.897	open-NTD-site10
J4E	−6.185	Gal-site1	KOZ	−6.894	open-NTD-site10
J4N	−6.169	Gal-site1	J3Q	−6.871	open-NTD-site10
DQT	−6.161	Gal-site1	KPB	−6.853	open-NTD-site10
KOE	−6.118	Gal-site1	KP8	−6.845	open-NTD-site9
J5Q	−6.091	Gal-site1	H5N	−6.838	open-NTD-site9
J5E	−6.082	Gal-site1	H5T	−6.828	close-NTD-site3
P8J	−6.061	Gal-site1	DQT	−6.797	open-NTD-site14
J5W	−6.061	Gal-site1	J5E	−6.752	open-NTD-site10
J3Q	−6.037	Gal-site1	BKK	−6.649	open-NTD-site4
J62	−5.946	Gal-site1	5SY	−6.586	close-NTD-site9
NAG	−5.089	Gal-site1	MQT	−6.144	close-NTD-site4
MQT	−5.062	Gal-site1	NAG	−5.548	close-RBD-site22
GCU	−4.701	Gal-site1	GCU	−5.114	open-NTD-site10
BEK	−4.517	Gal-site1	BEK	−4.883	close-RBD-site22
THR	−4.300	Gal-site1	THR	−4.668	close-NTD-site10
UNU	−3.798	Gal-site1	UNU	−4.275	close-NTD-site10

**Table 5:** Predicted toxicities and toxicity risk for 56 compounds performed by ADMET Predictor software. ADMET Predictor identifier of each toxicity is mentioned in the parenthesis. In particular, Tox_hERG_Filter is a qualitative estimation of the affinity to the hERG potassium channel in human and Tox_hERG is the affinity to the hERG potassium channel in human expressed as pIC50 in mol/L. Compounds with an IC50 less than or equal to 10 μmol/L were labelled Toxic (T), while those greater than 10 μmol/L are considered (NT). Human liver adverse effect (the likelihood of causing elevation in the levels of AlkPhos, GGT, LDH, AST and ALT enzymes) is also summarised in hepatotoxicity section and colour-coded as EL (Elevated), NL (Normal). Other toxicity assessments are mentioned in Skin sensitivity, Respiratory sensitivity, Reproductive toxicity, Phospholipidosis, Chromosome aberration, Estrogen and AndrogenToxicity and Max_RTD (Maximum Recommended Therapeutic Dose). The abbreviations used are Nonsensitive (NS), Sensitive (S), EL (Elevated), NL (Normal), T (Toxic) and NT (Nontoxic). Toxicity risk (possible range 0–7) is the risk connected with predicted toxicity problems a compound might have. Toxicity risk less than 2 is considered as safe. Check rules and abbreviations for TOX_Risk and ToX_Code in Section 2.6 for the codes. MV stands for MISSING_VALUE in the ADMET Predictor and colour-coded in yellow.

#	Compound Name	Skin sensitivity (Sens_Skin)	Respiratory Sensitivity (Sens_Resp)	Reproductive Toxicity (Repro_Tox)	Cardiotoxicity		Hepatotoxicity					Phospholipidosis (PLipidosis)	Chromosome Aberration (Chrom_Aberr)	Estrogen Toxicity (Estro_Filter)	Androgen Toxicity (Andro_Filter)	Max_RTD	Toxicity	
					(hERG_Filter)	IC50 [mol/L] (hERG_pIC50)	(Ser_AlkPhos)	(Ser_GGT)	(Ser_LDH)	Ser_AST)	(Ser_ALT)						Risk	Code
1	5KS	NS	NS	T	No	4014	EL	EL	NL	EL	EL	NT	NT	NT	T	Below_3.16	1	
2	5KT	NS	NS	T	No	4054	EL	EL	NL	EL	EL	NT	NT	NT	T	Below_3.16	1	Xr+; Xm+
3	5SY	NS	NS	NT	No	4795	EL	NL	NL	NL	EL	NT	T	NT	T	Above_3.16	1	Xr+; Xm-
4	DQT	NS	NS	T	No	4783	NL	EL	NL	NL	EL	NT	NT	NT	T	Below_3.16	1	Xr+; Xm-
5	MQT	NS	NS	T	No	4623	EL	EL	NL	NL	EL	NT	NT	NT	T	Above_3.16	1	Xr+; Xm-
6	9Q5	NS	NS	NT	No	4246	EL	EL	EL	EL	EL	NT	NT	NT	T	Below_3.16	2.64	rat; Xr+; Xm-; HEPX
7	9SK	NS	NS	T	No	4029	EL	EL	NL	EL	EL	NT	NT	NT	T	Below_3.16	3	hERG-; rat; Xr+; Xm-; HEPX-
8	BEK	NS	NS	T	No	4.71	EL	EL	NL	EL	EL	NT	NT	NT	NT	Above_3.16	2	Xr; Xm
9	EGZ	NS	NS	NT	No	3706	EL	EL	NL	EL	EL	NT	NT	NT	NT	Above_3.16	1.5	hERG-; Xr+; Xm-
10	8VT	NS	NS	NT	No	4344	EL	EL	NL	EL	EL	NT	NT	NT	T	Above_3.16	1	Xr+; Xm-
11	8VW	NS	NS	NT	No	4036	EL	EL	NL	EL	EL	NT	NT	NT	NT	Above_3.16	1.5	hERG-; Xr+; Xm-
12	P8J	NS	NS	NT	No	3575	EL	EL	NL	EL	NL	NT	NT	NT	NT	Above_3.16	1	Xr+; Xm-
13	H5T	NS	NS	NT	No	3955	EL	EL	NL	EL	EL	NT	NT	NT	NT	Below_3.16	2	hERG-; Xr-; Xm-; HEPX-
14	H5Z	NS	NS	T	No	4.23	EL	EL	NL	EL	EL	NT	NT	NT	T	Below_3.16	2	hERG-; Xr-; Xm-; HEPX-; MUT
15	H5Q	NS	NS	T	No	4434	EL	EL	NL	EL	EL	NT	NT	NT	T	Below_3.16	3	hERG-; Xr+; Xm-; HEPX-; MUT
16	H5N	NS	NS	T	No	4353	EL	EL	NL	EL	EL	NT	NT	NT	NT	Below_3.16	3	Xr-; Xm-; HEPX-; MUT
17	H5H	NS	NS	T	No	4205	EL	EL	NL	EL	EL	NT	NT	NT	T	Below_3.16	2.5	hERG-; Xr+; Xm-; MUT
18	P8G	NS	NS	NT	No	3238	EL	EL	NL	EL	NL	NT	NT	NT	NT	Below_3.16	2.5	hERG-; Xr+; Xm-; MUT
19	J5Q	NS	NS	T	No	4844	NL	EL	NL	EL	EL	NT	NT	NT	T	Below_3.16	1.5	Xr+; Xm
20	J3Q	NS	NS	T	No	4599	EL	EL	EL	EL	EL	NT	NT	NT	T	Below_3.16	2	Xr+; Xm-; HEPX
21	J5E	NS	NS	T	No	4762	EL	EL	NL	EL	EL	NT	NT	NT	T	Below_3.16	1	Xr+; Xm+
22	J4N	NS	NS	T	No	4768	EL	EL	NL	EL	EL	NT	NT	NT	T	Below_3.16	1	Xr+; Xm+
23	J4E	NS	NS	T	No	4.88	EL	EL	NL	EL	EL	NT	NT	NT	T	Below_3.16	1.5	Xr+; Xm
24	J5W	NS	NS	T	No	4828	EL	EL	NL	EL	EL	NT	NT	NT	T	Below_3.16	1.5	Xr+; Xm
25	HRK	NS	NS	T	No	4038	EL	EL	NL	EL	EL	NT	NT	NT	T	Below_3.16	1	Xr+; Xm+
26	J62	NS	NS	T	No	4887	EL	EL	NL	EL	EL	NT	NT	NT	T	Below_3.16	1.5	Xr+; Xm
27	UNU	NS	S	T	No	4774	EL	EL	EL	EL	EL	NT	T	NT	NT	Above_3.16	1	HEPX
28	THR	NS	NS	NT	No	4343	NL	EL	EL	NL	NL	NT	NT	NT	NT	Above_3.16	0	
29	E61	NS	NS	T	No	3398	EL	NL	NL	EL	EL	T	NT	NT	NT	Below_3.16	2.88	hERG-; rat; Xr+; Xm-; HEPX-
30	BKH	NS	S	T	No	4877	EL	EL	EL	EL	EL	NT	NT	NT	T	Below_3.16	2	Xr+; Xm-; HEPX
31	BKK	NS	NS	T	No	4.79	EL	EL	EL	EL	EL	NT	NT	NT	T	Below_3.16	2	Xr+; Xm-; HEPX
32	TD2	NS	NS	T	No	4	EL	EL	NL	EL	EL	NT	NT	NT	T	Below _3.16	1	Xr+; Xm+
33	TGZ	NS	NS	T	No	4.03	EL	EL	NL	EL	EL	NT	NT	NT	T	Below_3.16	1	Xr+; Xm+
34	J1E	NS	NS	T	No	4585	EL	EL	NL	EL	EL	NT	NT	NT	T	Below_3.16	1	Xr; Xm; Xr+; Xm+
35	KP8	NS	NS	T	No	4537	EL	EL	EL	EL	EL	NT	NT	NT	T	Below_3.16	1.5	Xr+; Xm-; HEPX+
36	KOZ	NS	NS	T	No	4.79	EL	EL	EL	EL	EL	NT	NT	NT	T	Below_3.16	2	Xr+; Xm-; HEPX
37	KPB	NS	NS	T	No	4511	EL	EL	EL	EL	EL	NT	NT	NT	T	Below_3.16	1.5	Xr+; Xm-; HEPX+
38	KON	NS	NS	T	No	4607	EL	EL	EL	EL	EL	NT	NT	NT	T	Below_3.16	2	Xr-; Xm-; HEPX
39	KOE	NS	NS	T	No	4724	EL	EL	EL	EL	EL	NT	NT	NT	T	Below_3.16	2	Xr+; Xm-; HEPX
40	J0T	NS	NS	T	No	4585	EL	EL	NL	EL	EL	NT	NT	NT	T	Below_3.16	1	Xr; Xm; Xr+; Xm+
41	A6J	NS	NS	NT	No	3248	EL	NL	NL	EL	EL	NT	NT	NT	NT	Below_3.16	2	hERG-; Xr+; Xm-; HEPX-
42	GMK	NS	S	NT	No	3898	EL	NL	NL	NL	NL	NT	NT	NT	NT	Above_3.16	1.5	Xr; Xm; hERG-; Xr+; Xm-
43	70B	NS	NS	T	No	4.08	EL	EL	NL	NL	EL	NT	NT	NT	T	Above_3.16	1.5	Xr; Xm; hERG-; Xr+; Xm-
44	KOW	NS	NS	T	No	4.03	EL	EL	NL	EL	EL	NT	NT	NT	T	Below_3.16	1	Xr+; Xm+
45	KP5	NS	NS	T	No	4011	EL	EL	NL	EL	EL	NT	NT	NT	T	Below_3.16	1	Xr+; Xm+
46	KOQ	NS	NS	T	No	4036	EL	EL	NL	EL	EL	NT	NT	NT	T	Below_3.16	1	hERG; Xr; Xr+; Xm+
47	GCU	NS	NS	NT	No	4024	EL	EL	NL	EL	EL	NT	NT	NT	NT	Above_3.16	1	Xr+; Xm-
48	NAG	NS	NS	NT	No	4147	EL	EL	EL	EL	EL	NT	NT	NT	NT	Above_3.16	2	Xr+; Xm-; HEPX
49	NEW1	NS	NS	T	No	3.854	EL	EL	EL	EL	EL	NT	NT	NT	T	Below_3.16 (71%)	1.5	Xr+; Xm+; HEPX+
50	NEW2	NS	NS	T	No	3.649	EL	EL	NL	EL	EL	NT	NT	NT	T	Below_3.16	2	hERG-; Xr+; Xm-; HEPX-
51	NEW3	NS	NS	T	No	3.916	EL	EL	EL	EL	EL	NT	NT	NT	T	Below_3.16 (71%)	2	Xr+; Xm+; HEPX
52	NEW4	NS	NS	NT	No	3.589	EL	NL	EL	EL	NL	NT	NT	NT	NT	Below_3.16 (91%)	1.5	hERG-; Xr+; Xm-
53	NEW5	NS	NS	NT	No	3.565	EL	NL	NL	NL	NL	NT	NT	NT	NT	Above_3.16 (59%)	1.5	hERG-; Xr+; Xm-
54	NEW6	NS	NS	T	No	4.022	EL	EL	EL	EL	EL	NT	NT	NT	T	Below_3.16 (71%)	1.5	Xr+; Xm-; HEPX+
55	NEW7	NS	NS	T	No	3.779	EL	EL	NL	EL	EL	NT	NT	NT	T	Below_3.16	2	hERG-; Xr+; Xm-; HEPX-
56	NEW8	NS	NS	T	No	4.078	EL	EL	EL	EL	EL	NT	NT	NT	T	Below_3.16 (71%)	2	Xr+; Xm-; HEPX

**Table 6:** Amino acid mutations of SARS-CoV-2 Alpha, Beta, Gamma and Delta variants of SARS-CoV-2 with a focus on Spike protein. The domain to which each mutation belongs is indicated in parentheses.

B.1.1.7 (United Kingdom, Alpha)	B.1.351 (South Africa, Beta)	P.1 (Brazil, Gamma)	B.1.617.2 (India, Delta)
H69–V70 deletion (NTD)	L18F (NTD)	L18F (NTD)	T19R (NTD)
Y144 deletion (NTD)	D80A (NTD)	T20N (NTD)	157-158 Deletion (NTD)
N501Y (RBD)	D215G (NTD)	P26S (NTD)	L452 (RBD)
A570D	242-244 deletion (NTD)	D138Y (NTD)	T478(RBD)
P681H	R246I (NTD)	R190S (NTD)	D614G
T716I	K417N (RBD)	K417T (RBD)	P681R
S982A	E484K (RBD)	E484K (RBD)	D950N
D1118H	N501Y (RBD)	N501Y (RBD)	
	D614G	D614G	
	A701V	H655Y	
		T1027I	
		V1176F	

## Data Availability

The datasets used and analysed in the current study are available from the corresponding author upon request.

## Supplementary Materials

The following are available online at https://www.mdpi.com/article/10.3390/biomedicines9091208/s1, Figure S1: 2D chemical structures of the screened ligands along with their names.
