# Supplementary material for: Computational Study of Potential Galectin-3 Inhibitors in the Treatment of COVID-19
Source: Biomedicines. 2021 Sep 13;9(9):1208. doi: 10.3390/biomedicines9091208 (PMC8466820; doi:10.3390/biomedicines9091208)
Supplement: Supplementary file 1 [file biomedicines-09-01208-s001.zip › biomedicines-1378665-SI.pdf]

**name:** 5KS 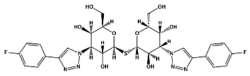	**name:** 5KT 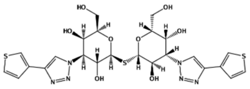	**name:** 5SY 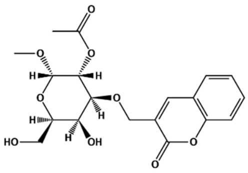	**name:** DQT 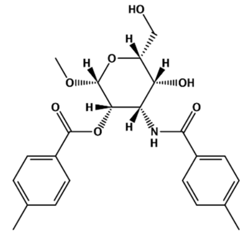
**name:** MQT 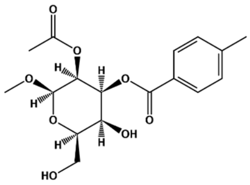	**name:** 9Q5 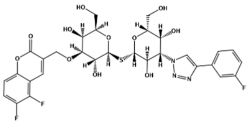	**name:** 9SK 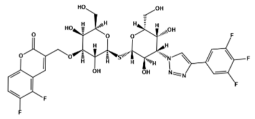	**name:** BEK 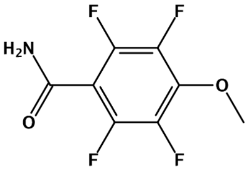
**name:** EGZ 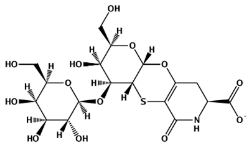	**name:** 8VT 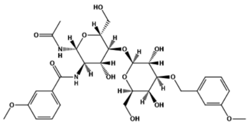	**name:** 8VW 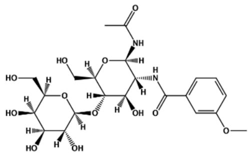	**name:** P8J 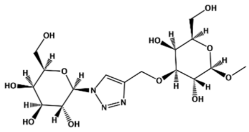
**name:** H5T 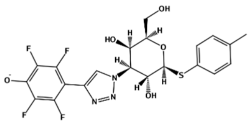	**name:** H5Z 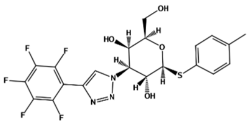	**name:** H5Q 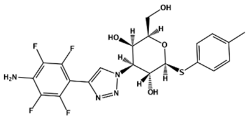	**name:** H5N 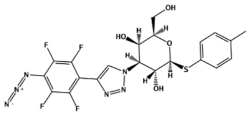
**name:** H5H 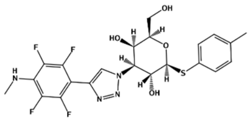	**name:** P8G 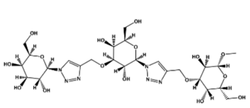	**name:** J5Q 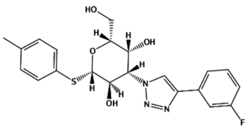	**name:** J3Q 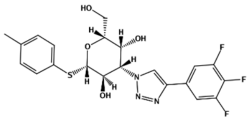

**name:** J5E 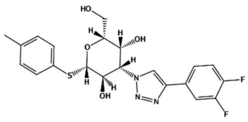	**name:** J4N 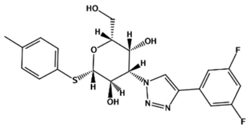	**name:** J4E 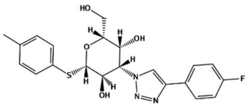	**name:** J5W 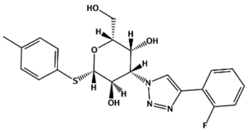
**name:** HRK 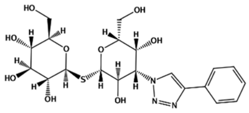	**name:** J62 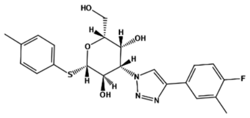	**name:** THR 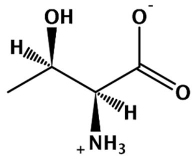	**name:** E61 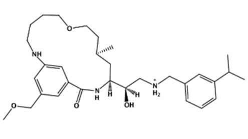
**name:** BKH 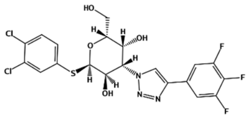	**name:** BKK 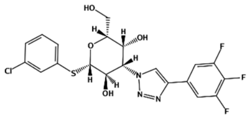	**name:** TD2 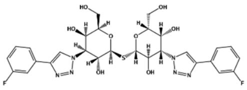	**name:** TGZ 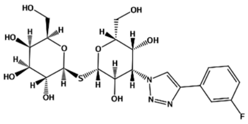
**name:** J1E 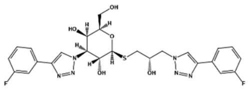	**name:** KP8 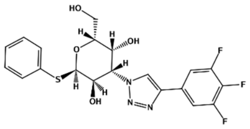	**name:** KOZ 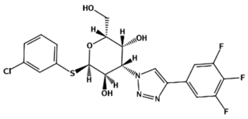	**name:** KPB 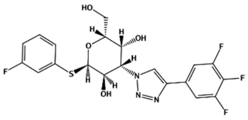
**name:** KON 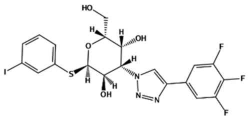	**name:** KOE 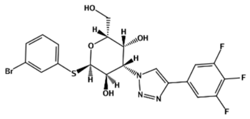	**name:** J0T 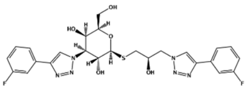	**name:** A6J 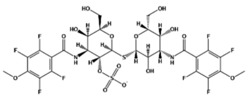

**name:** GMK<br>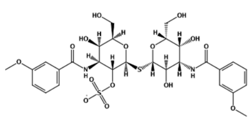	**name:** 70B<br>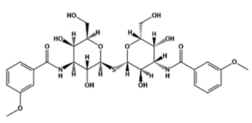	**name:** KOW<br>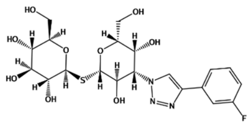	**name:** KP5<br>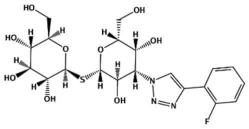
**name:** KOQ<br>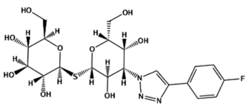	**name:** GCU<br>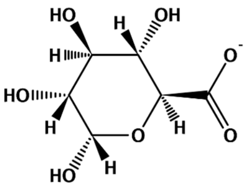	**name:** NAG<br>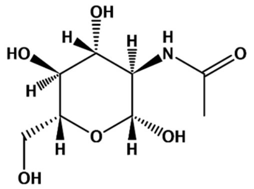	**name:** NEW6<br>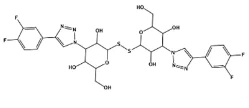
**name:** NEW7<br>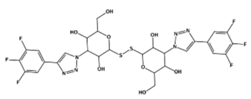	**name:** NEW8<br>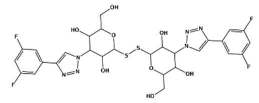	**name:** NEW4<br>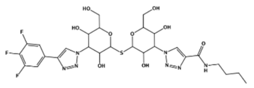	**name:** NEW5<br>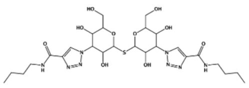
**name:** UNU<br>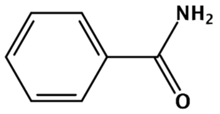	**name:** NEW1<br>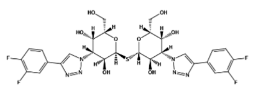	**name:** NEW3<br>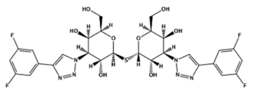	**name:** NEW2<br>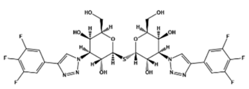

Figure S1. 2D chemical structures of the screened ligands along with their names
